# Liver Injury in Favipiravir-Treated COVID-19 Patients: Retrospective Single-Center Cohort Study

**DOI:** 10.3390/tropicalmed8020129

**Published:** 2023-02-20

**Authors:** Amal Oweid Almutairi, Mahmoud Zaki El-Readi, Mohammad Althubiti, Yosra Zakariyya Alhindi, Nahla Ayoub, Abdullah R. Alzahrani, Saeed S. Al-Ghamdi, Safaa Yehia Eid

**Affiliations:** 1 Department of Pharmacology and Toxicology, Faculty of Medicine, Umm Al-Qura University, Al Abdeyah, Makkah 24381, Saudi Arabia; 2 Saudi Toxicology Society, Umm Al-Qura University, Makkah 24381, Saudi Arabia; 3 Clinical Pharmacy, General East Jeddah Hospital, Jeddah 22253, Saudi Arabia; 4 Department of Biochemistry, Faculty of Medicine, Umm Al-Qura University, Al Abdeyah, Makkah 24381, Saudi Arabia; 5 Biochemistry Department, Faculty of Pharmacy, Al-Azhar University, Assuit 71524, Egypt

**Keywords:** COVID-19, favipiravir, liver injury

## Abstract

(1) Background: Favipiravir (FVP) is a new antiviral drug used to treat COVID-19. It has been authorized to be used in the kingdom of Saudi Arabia in the treatment of COVID-19. The mechanism of action of FVP is working as a specific inhibitor for the RNA-dependent RNA polymerase of the RNA chain virus. FVP has the potential to be hepatotoxic because of the structure similarity with pyrazinamide. This retrospective study aimed to determine the prevalence of liver injury in FVP-treated COVID-19 patients in General East Jeddah Hospital, Saudi Arabia, during the COVID-19 pandemic. (2) Methods: A total of 6000 patients infected with COVID-19 and treated at the East Jeddah Hospital were included, with a sample size of 362 patients. The participants ranged from 18 to 70 years of age, both males and females, with normal hepatic and renal function and had a confirmed diagnosis of COVID-19 infection. Patients who had gouty arthritis, hepatic and renal dysfunction, dead patients, pregnant women, and breastfeeding mothers were all excluded from this study. A retrospective cohort study compared two groups of patients treated with and without FVP and who followed the Saudi Ministry of Health protocol to manage COVID-19 infection. (3) Results: An adverse effect of FVP on the liver was found that ranged from mild to severe. Stopping treatment with FVP was associated with an observed important increase in the levels of liver enzymes AST (*p* < 0.001), ALT (*p* < 0.001), alkaline phosphatase (*p* < 0.03), total bilirubin (*p* < 0.001), and direct bilirubin (*p* < 0.001) in the treated compared with the untreated group. (4) Conclusion: This study showed a significant difference between the treated and the untreated groups with FVP in liver injury. FVP influences the liver, increasing the blood levels of the liver function parameters.

## 1. Introduction

There has been a considerable increase in the spread of a new type of novel variant coronavirus infection (COVID-19) worldwide since December 2019 [1]. COVID-19 primarily affects the respiratory system and can cause asymptomatic to mild or severe illness requiring ICU admission [2]. The elderly and individuals with comorbidities such as hypertension, diabetes mellitus, malignancy, etc., are at high risk of having severe symptoms [3]. The most frequent manifestations of COVID-19 infection are increased body temperature, cough, shortness of breath, lethargy, loss of appetite, gastrointestinal disturbance, flu-like symptoms, tiredness, irritation of the throat, headache, and inflammation of the outermost layer of the white part of the eye and the inner surface of the eyelid [4,5].

The possible seriousness of COVID-19 and its lethal complexities require rushing the evolution of medications to stop the spread and manage the Coronavirus. Although there is no known medication for COVID-19 management, ongoing research and clinical preliminary studies are being conducted to investigate the ability of multiple drugs to be reused to treat COVID-19 infection. The available medicines reused in the treatment of COVID-19 are many, such as immunomodulatory drugs (tocilizumab), adjunctive therapy (antibiotics, corticosteroids), and antiviral drugs such as FVP, oseltamivir, and Kaletra, given orally and parenterally as Remdesivir [6].

FVP is an antiviral medication with a wide range of activity against influenza viruses such as arena-, bunya-, flavor-, and filoviruses and has been approved for use in COVID-19-infected patients in Saudi Arabia and many other countries. FPV is an inactive drug that needs activation to act and passes inside the cells by endocytosis, where the absorption mechanism becomes active. FVP is activated by phosphorylation and phosphoribosylation to produce the FVP ribofuranosyl-triphosphate that suppresses viral replication by focusing on RNA-dependent RNA polymerase [7,8,9]. FVP is given orally with 1600–1800 mg twice per day as a loading dose, followed by 600–800 mg twice per day for 7–10 days [10]. According to pharmacokinetics, FVP has good bioavailability (94%), a low volume of distribution, a short half-life ranging from 2.5 to 5 hours, and is highly bound to albumin. It is excreted via the renal pathway, has no effect on cytochrome P450, and acts as a CP2C8 inhibitor [11].

The most common adverse effects of FVP are an increase in uric acid levels in the blood, diarrhea, elevation of liver enzymes, hyperemesis, and skin problems such as dermatitis and palpitations [12,13].

One of the medications used in managing COVID-19 infection in the Saudi Ministry of Health guidelines and protocol is the antiviral FVP, which has been accepted in many countries for COVID-19 treatment [7,14,15].

Pramod Kumar has described the side effects of FVP on the liver by publishing a case report for three patients who had abdominal pain and complained about changes in their skin color, which became yellow. Aspartate transaminase, alanine transaminase, and alkaline phosphatase levels were increased. All three patients had a routine liver laboratory investigation before FVP treatment initiation and were treated with the same dose (1800 mg twice per day on the first day as a loading dose, then 800 mg twice daily as a maintenance dose for 10–14 days). The causes of liver impairment were excluded, such as medications, alcohol, or liver disease. The liver injury was measured using the Roussel-Uclaf causality assessment method with a score of 7. They suspected that the liver injury was caused by FVP doses used to treat COVID-19 infection or by a FVP active metabolite [16].

The systemic review and meta-analysis for FVP’s safety compared patients treated and not treated with FVP. This meta-analysis included nine studies that differ in the study design and all other variables such as ethnicity, age, etc. One of the objectives was to evaluate the adverse effects. They noted no significant difference in side effects between the FVP and control groups. Still, they found that FVP has many negative consequences, one of which is an elevation in liver enzymes [17].

Another study analyzed the chances of liver injury for all drugs used to manage COVID-19, and one of the medications is FVP. However, they explained that there was insufficient information about liver injury for FVP [18]. Another study found that there was an elevation in liver enzymes such as alanine transaminase and aspartate transaminase, which indicated cellular injury in FVP-treated COVID-19 patients [19]. 

Based on the chemical structure, FVP may also be prone to induce liver damage. Hepatotoxicity is a common side effect of the antituberculosis medication pyrazinamide, while the precise mechanism is unknown [20]. FVP is a medicine that has the potential to be hepatotoxic because of how structurally similar it is to pyrazinamide. In mild to moderate COVID-19 patients without a history of liver disease, 5 days of FVP therapy did not significantly influence the liver enzymes; only brief elevations occurred [21].

Although the precise cause of the liver damage is unknown, it is possible that FVP or one of its metabolites caused the injury. Additionally, we hypothesize that liver damage may result at a higher dose. Additionally, prolonged use inhibits its liver metabolism, which could raise the ratio of FVP to an inactive metabolite [16].

The main side effects of FVP are regarded as teratogenicity and hyperuricemia, but less is known about other potential side effects, such as drug-induced liver injury [16,20,21]. This retrospective study aimed to determine the prevalence of liver injury in FVP-treated COVID-19 patients in General East Jeddah Hospital, Saudi Arabia, during the COVID-19 pandemic.

## 2. Research Design and Methods

A retrospective cohort study was conducted to evaluate the liver injury caused by FVP. Permission to enter the health information system for my medical report to use the patient’s information and laboratory section was obtained. The registration number with KACST and KSA was H-02-J-002, and the Umm Al-Qura University registration number in the National Committee of Bio-Ethics was HAPO-02-K-012. The collected data from the hospital information system (careware) were added to an Excel datasheet. Patients with COVID-19 were studied at Jeddah’s East Jeddah hospital from May to August 2020.

The inclusion criteria for this study were patients with an age ranging from 18 to 70 years, with normal liver and renal function, of both genders and nationalities, and with a confirmed diagnosis of COVID-19 infection. The exclusion criteria were known cases of gouty arthritis, renal and hepatic dysfunction, pregnant and breastfeeding women, and dead patients. The liver injury of FVP was assessed based on liver function parameters: alanine aminotransaminase, total bilirubin, direct bilirubin, alkaline phosphatase, and aspartate aminotransaminase, by measuring their blood levels. Before beginning treatment with any medications and after beginning therapy with FVP or COVID-19 protocol medications, the liver function parameters were collected [22].

The liver injury caused by the drug was defined as a liver biochemical abnormality and a liver biopsy that has not been performed. The biochemical irregularity was characterized by blood level elevations of alanine transaminase, aspartate transaminase, total bilirubin, direct bilirubin, and alkaline phosphatase at two times the highest limit or five times the limit without symptoms [23].

The study sample was divided into two groups; one was treated with FVP 1800 mg twice for one day, followed by 800 mg twice daily for 7–10 days, while the other was not. The Saudi ministry of health protocol followed all two groups to manage COVID-19 infection [15].

### 2.1. Sample Size and Sample Selection

The total population included 6000 patients, and the sample size was 362, which was calculated online using Qualtrics [24] to achieve a confidence interval of 95% and a margin of error of 5%. Then, the 362 were divided into two groups. Each group contained 181, which was collected randomly from the excel datasheet and then added to another excel sheet for statistical analysis.

### 2.2. Data Collection

A pre-designed checklist was prepared to collect data about patients’ demographics, chronic diseases, medications used, and liver function enzymes.

### 2.3. Statistical Analysis

The following statistical tests were used, and all data were collected, calculated, tabulated, and statistically analyzed. A normality test (Kolmogorov–Smirnov) was performed to check the normal distribution of the samples. Descriptive statistics were computed using Mean ± Standard deviation (SD). Data were represented as numbers and percentages (%) for categorical variables, median, and mean for the variables measured using indicators. The chi-square test was used to evaluate the association between qualitative data. Paired and unpaired sample *t*-tests were used to compare every two groups before and after treatment. The Pearson correlation coefficient was used to assess the correlations between variables. This helpful technique analyzes the relationship between a single dependent variable and several independent variables. One-way ANOVA was used to compare age categories for each liver enzyme. A *p*-value less than 0.05 was considered statistically significant. All statistical analyses were performed using the computer program SPSS software for Windows version 26.0 (Statistical Package for Social Science, Armonk, NY, USA: IBM Corp) at a significant level of less than 0.05 (*p*-value < 0.05).

## 3. Results

### 3.1. Demographic Profile and General Information for All Patients under Study

This study included 362 COVID-19 patients in a single center in Jeddah city called General East Jeddah Hospital. There were two groups; the first group (181 patients) received FPV and the second did not (181). Table 1 shows the baseline characteristics of the population samples. Most of the patients were males, 275 (76.0%), while there were 87 females (24.0%). Regarding nationalities, most of the patients were non-Saudi, with a total number of 264 (72.9%), while Saudis were 98 (27.1%). The age ranged from 24 to 67 years, with a mean age of 51.29 ± 10.43 years. Most of the patients were in the age category of 40 to 60 years (middle age), in which there were 204 patients (56.4%), followed by >60 years (elderly), 92 (25.4%); while the younger patients <40 years (adult) totaled 66 (18.2%). A comparison between genders, nationalities, and age categories among treated and untreated patients with FPV is presented in Table 1.

### 3.2. Description of Chronic Diseases of the Participating Patients

**Figure 1:**
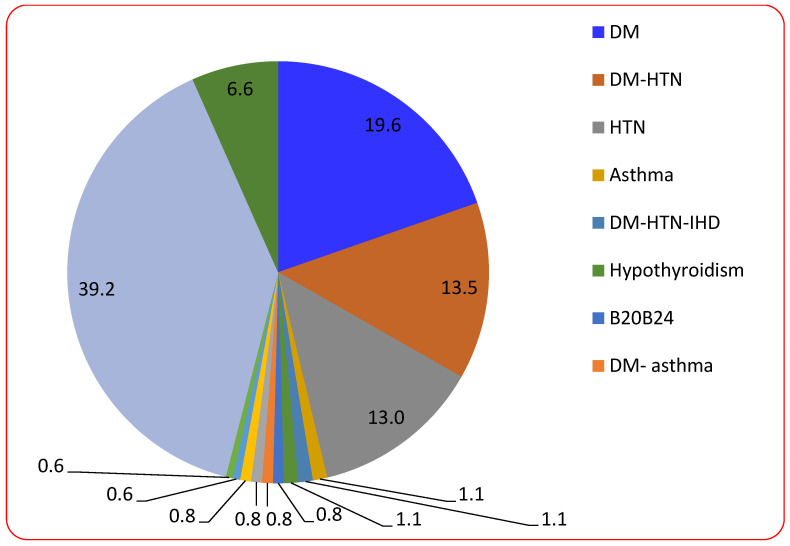
The most important chronic diseases of the participating patients.

Figure 1 shows this study’s most important and most frequent chronic diseases. It was found that the most frequent chronic disorders were DM (about 19.6%), HTN (13.0%), and both DM and HTN (13.5%). As shown below, asthma, hypothyroidism, and dyslipidemias were approximately 1.1% of the total patients and about 6.6% of patients with other chronic diseases.

### 3.3. Most of the Medicines Are Taken by the Patients under Study

**Figure 2:**
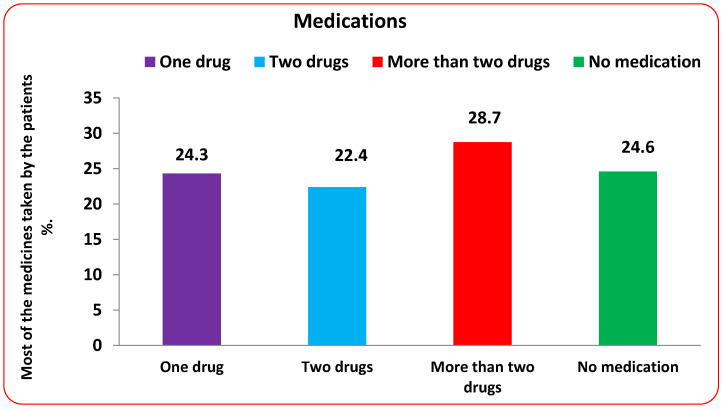
The mono, dual, and multiple therapies of the studied patients.

Medications used are illustrated in Figure 2, which shows that about 24.3% of the patients took one drug, and the most used drug was Amlodipine. About 22.4% were taking two drugs, and the most used of them was Novo rapid—Lantus; the most significant proportion of the total patients were taking more than three drugs, and about 28.7% (Lantus—Novo rapid—Amlodipine, Sitagliptin—Amlodipine—Metformin, and Valsartan—Hydrochlorothiazide—Novo rapid—Lantus) were considered using more than two drugs. About 24.6% of the patients in this study were not taking any medications other than the Saudi Ministry of Health protocol for COVID-19 management (Figure 2).

### 3.4. The Effect of Using Another Medication on Liver Enzymes

The results in Table 2 show the effect of drugs on liver enzymes compared to patients who did not receive any medications for the treatment of chronic disease. Statistical analysis showed a non-significant increase in almost all liver function parameters in the patients who did not receive medications and those who received medications for all parameters using the independent *t*-test at a *p*-value <0.05.

### 3.5. Comparison between Genders, Nationality, and Age Categories in Treated and Untreated with FPV

**Figure 3:**
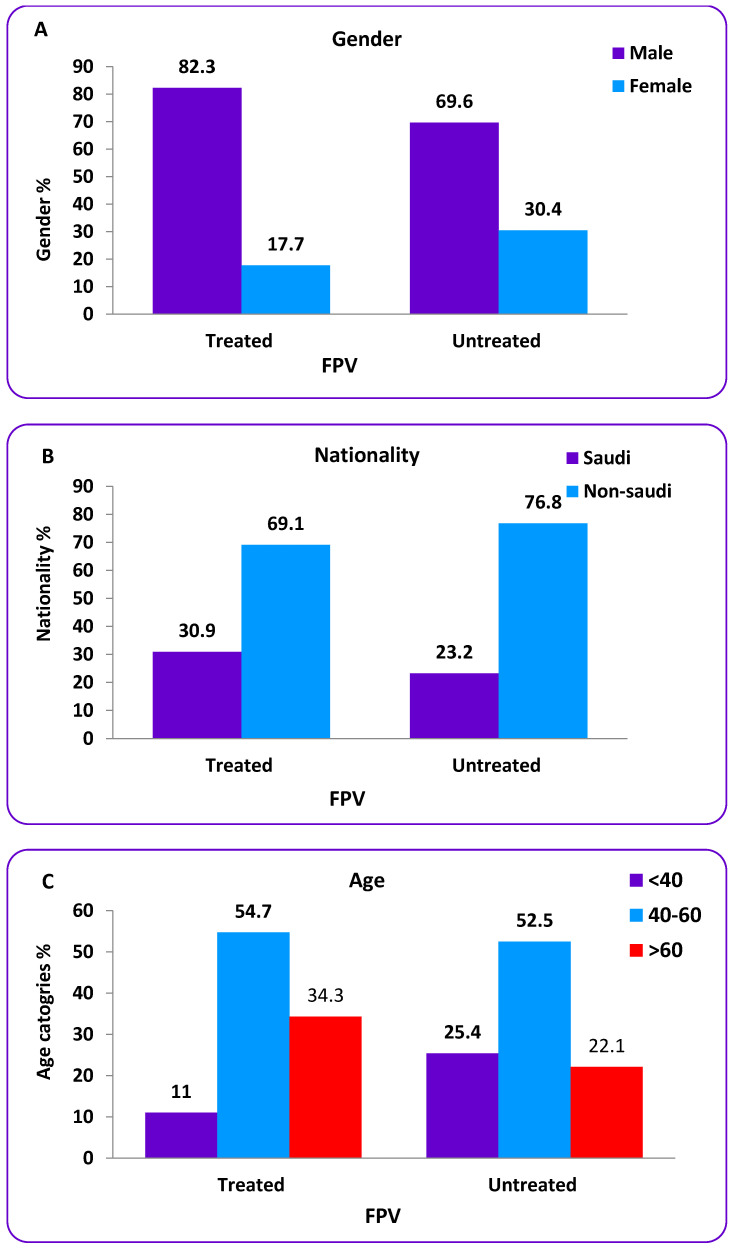
Frequency ratio of males and females (**A**), nationalities (**B**), and ages (**C**) in treated and untreated patients with FPV.

The results show that there was a statistically significant gender difference between treated and untreated patients with FPV using the chi-square test (*p* < 0.001) as most of the patients were males, 149 (82.3%) and 126 (69.6%), while females were 32 (17.7%) and 55 (30.4%) in treated and non-treated groups, respectively. Regarding ethnicity, statistical analysis showed a non-significant difference between treated and non-treated patients using the chi-square test (*p* = 0.09) Figure 3. According to nationality, most of the patients were non-Saudi 125 (69.1%) and 139 (76.8%), while patients from Saudi Arabia were 56 (30.9%) and 42 (23.2%) in treated and non-treated groups, respectively. For age categories, there was a statistically significant difference between age categories using the chi-square test at (*p* < 0.001), as most of the patients participating in this study were between the ages of 40 and 60 years for the two groups Figure 3.

### 3.6. Pearson Correlation Coefficient between the Liver Functions (before and after FPV Treatment)

Results in Table 3 show the Pearson correlation coefficient (r values, *p*-value) between the liver functions before and after treatment with FPV. Regarding the correlation coefficient between the different functions of the livers for all the patients before taking FPV, it was found that the AST had a significant positive correlation with ALT (r = 0.440) and with direct bilirubin (r = 0.240). As for the Alt, it had a positive significant correlation with alkaline phosphates (r = 0.235), total bilirubin (r = 0.244), and direct bilirubin (r = 0.292). Alkaline phosphatase had a significant positive correlation with total bilirubin (r = 0.187) and direct bilirubin (r = 0.233). It was also found that there was a significant positive correlation between the total bilirubin and direct bilirubin with r = 0.526 at *p* < 0.01. On the other hand, for the liver functions of the patients after treatment with FPV, the AST had a significant positive correlation with ALT (r = 0.369), total bilirubin (r = 0.259), and direct bilirubin (r = 0.325), while simultaneously, the ALT levels had a positive non-significant correlation with other liver functions. Alkaline phosphatase had a negative non-significant correlation with the total bilirubin (r = −0.023) and direct bilirubin (r = −0.073). At the same time, the total bilirubin had a positive, highly significant correlation with direct bilirubin with r = 0.891 at *p* < 0.01.

### 3.7. Comparison between before and after According to Treated and Untreated FPV Therapy

**Figure 4:**
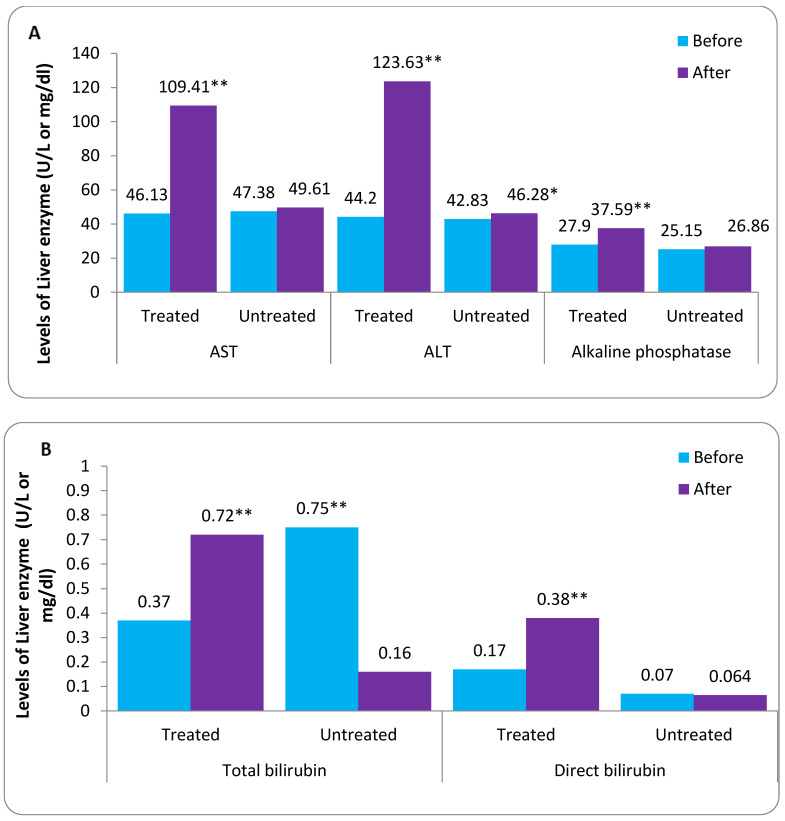
Comparison between before and after according to treated and untreated FPV Therapy (**A**,**B**). * Significant difference using paired *t*-test at *p* < 0.05 and ** at *p* < 0.01.

The liver function enzymes AST, ALT, alkaline phosphatase, and bilirubin levels at the end of the study in treated and untreated groups are shown in Table 4. The statistical analysis showed a significant difference before and after FPV therapy for AST, ALT enzyme, alkaline phosphatase, total bilirubin, and direct bilirubin in the treated group with FPV therapy. On the other hand, all the patients in the untreated group showed no significant difference for all variables using the paired sample t-test at *p* < 0.05 (Figure 4). However, the results show that there was an increase (% change) in the levels of liver enzymes (AST and ALT), alkaline phosphatase, total bilirubin, and direct bilirubin in treated groups by 137.18%, 72.17%, 34.73%, 94.59%, and 123.53%, respectively, which was about 4.71%, 8.05%, 6.80%, 78.67%, and 8.57% in the untreated group with FPV overall patients (*n* = 361).

### 3.8. Comparison between Treated and Untreated after FPV Therapy

The liver functions enzymes AST, ALT, alkaline phosphatase, and bilirubin levels at the end of this study after FPV therapy in treated and untreated groups are shown in Table 5. The statistical analysis shows a significant difference between treated and untreated according to AST, ALT enzyme, alkaline phosphatase, total bilirubin, and direct bilirubin levels using the unpaired sample *t*-test at *p* < 0.05. However, results show an increase in liver enzymes (AST and ALT), alkaline phosphatase, total bilirubin, and direct bilirubin in the treated compared with the untreated group.

### 3.9. Comparison between Patients Who Were Treated with FVP Based on Chronic Disease

**Figure 5:**
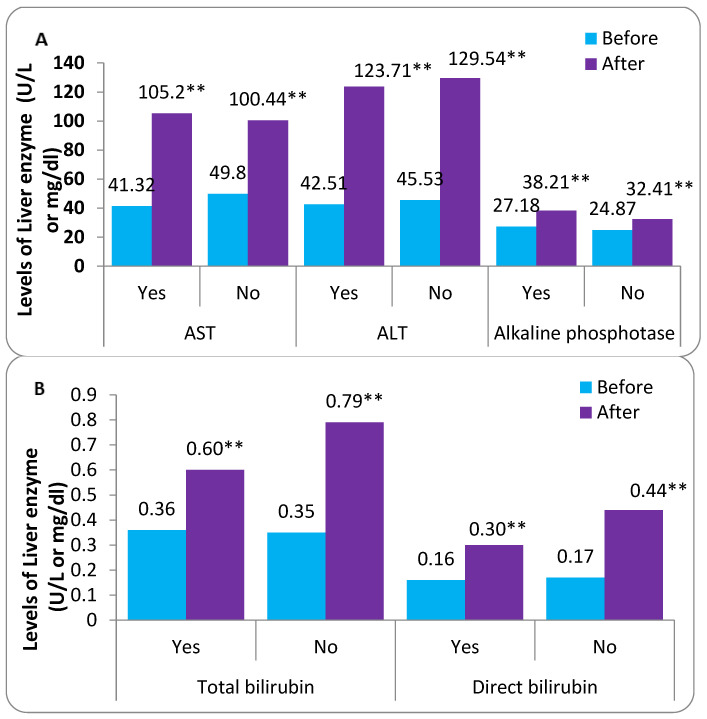
Comparison between Patients who were treated with FVP according to Chronic Disease (**A**,**B**). ** means significant difference using paired *t*-test at *p* < 0.05.

The results in Figure 5 show the differences in the different parameters (liver enzymes) between the cases with chronic diseases and healthy cases and those who received treatment with FVP before and after treatment. The statistical analysis showed statistically significant differences for all patients under the study according to all parameters (AST, ALT, alkaline phosphatase, total bilirubin, and direct bilirubin) before and after treatment at *p* < 0.05. At the same time, there are no statistically significant differences between the cases with chronic diseases and healthy subjects according to all parameters except for AST before treatment only.

### 3.10. Comparison between Patients Treated and Untreated with FVP Based on Chronic Disease

The results in Table 6 show the differences in the different parameters (liver enzymes) between the cases with chronic diseases and healthy case subjects and those who did not receive FVP before and after treatment. Statistical analysis showed no statistically significant differences for all claims under study for all parameters (AST, ALT, alkaline phosphatase, total bilirubin, and direct bilirubin) in patients with chronic diseases and healthy subjects for all parameters except the patients in AST before treatment. On the other hand, statistical analysis showed a statistically significant difference between all parameters before and after treatment using the paired *t*-test at *p* < 0.05.

### 3.11. Effect of Age Categories on Liver Function

**Figure 6:**
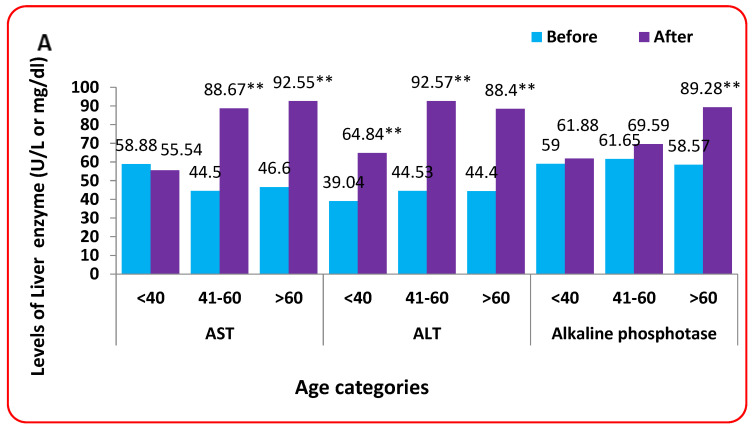

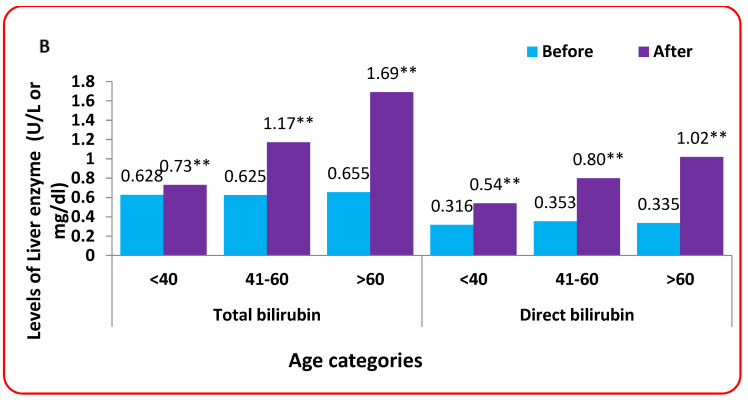
Effect of age categories on liver function (**A**,**B**). ** means significant difference using paired *t*-test at *p* < 0.05.

Regarding AST, it was found that there were significant differences between different age categories before and after FPV. However, it was also found that AST increased in patients in the 40–60 years category and patients with an age of more than 60 years. For ALT, alkaline phosphatase, total bilirubin, and direct bilirubin, there was a significant difference between different age categories in the patients after FPV treatment only. In addition, the results show a substantial difference between the patients before and after FPV treatment, except for alkaline phosphatase at the age of less than 40 years and 40–60 years. Generally, it was found that enzymes increased significantly in the patients after treatment with FPV compared to their levels before FPV for all age categories (Figure 6A,B).

## 4. Discussion

The coronavirus has spread quickly since its first recognizable reported case in patients with severe pneumonia in Wuhan, China. Close communication, airborne droplets, and potential aerosol transmissions are infection pathways for COVID-19 [25].

COVID-19 affects the respiratory system and can cause systemic manifestations such as gastrointestinal and hepatic symptoms. Liver injury has been seen in many patients, particularly those with serious illnesses. Patients with known persistent liver illnesses and those without previous liver problems could be at an increased risk of liver dysfunction. The worsening of the liver function indicates a poor prognosis. Patients who develop liver damage because of the coronavirus are typically men, the elderly, and the obese [26].

Lactate dehydrogenase, aspartate aminotransferase (AST), alanine transaminase (ALT), C-reactive protein, creatine kinase, erythrocyte sedimentation rate, white blood cell count, D-dimer level, procalcitonin, urea, and creatinine levels have all increased in clinical laboratory studies. COVID-19 patients had reduced hemoglobin, lymphocyte count, eosinophil count, and serum albumin values [27].

COVID-19 identifies direct viral cytopathologic injury and secondary liver impairment. This was attributed to the systemic inflammatory response or hypoxia, drug-induced liver failure, and ultimately the worsening of chronic liver illnesses as possible etiologic causes for liver injury. Angiotensin-converting enzyme 2 (ACE2) appears to be a significant player in COVID-19-induced liver damage [26].

There has previously been no definitive antiviral treatment for Coronavirus. In this way, identifying different drug treatment options is essential for controlling and managing the coronavirus flare-up. Obtaining Emergency Use Authorization from the Food and Drug Administration for newly available drugs or developing new antivirals against COVID-19 infection is one of the quickest ways to observe treatment [28]. Previously, it has been reported that in critically ill COVID-19 patients, comorbidities and hyper-responsive inflammatory or immunological indicators can predict how bad the illness will get as it becomes worse in the ICU. During the height of the pandemic, RVP and ipi showed little promise in improving biomarkers of prognosis, survival rate, or disease progression. However, emergency use authorization and repurposing the usage of different antivirals are being tested [29].

A medication-induced liver injury is a lesion or dysfunction of the liver caused by the medication. It could result in abrupt liver failure and the need for a liver transplant. Because almost all the medicines used to treat COVID-19, such as oseltamivir, lopinavir/ritonavir, ribavirin, chloroquine phosphate, and hydroxyl chloroquine sulfate, are metabolized in the liver, liver damage and an increase in liver enzymes are expected. Lipophilicity, mitochondrial liability, the formation of reactive metabolites, the metabolism pathway in the liver, and the potential to inhibit hepatic transporters are essential aspects that might lead to hepatotoxicity in vulnerable hosts [18].

Patients who developed FVP-induced liver damage were found to have greater FVP serum levels than those who did not. Increased ALT, AST, ALP, and total bilirubin were found to occur after using FVP. ALT increases with FVP administration and occurs in approximately 10% of patients with COVID-19 infection [18].

Recent pathological findings from COVID-19 revealed substantial microvascular steatosis and modest lobular inflammation, implying that drug-induced liver injury may be present [30].

Although no effective antiviral medicines for COVID-19 have been identified, antiviral therapies such as oseltamivir, arbidol, lopinavir, and ritonavir may cause abnormal liver function [30].

One of the antiviral medications used in COVID-19 management is FPV [31]. Because of the limited published evidence about FPV safety in COVID-19 treatment, the current retrospective single-center cohort research aimed to assess FPV’s safety for the liver in managing COVID-19 cases in Saudi Arabia, specifically in Jeddah’s General East Jeddah Hospital.

There were two groups of COVID-19 patients in this retrospective cohort: one was treated with FVP, while the other was treated with standard care to compare the efficacy of FVP [31]. In our study, we followed the same study design as the retrospective cohort study but evaluated the liver injury caused by FVP.

A review of past patients with COVID-19 who had their serum FVP trough concentration monitored during treatment under steady-state conditions was conducted. On the first day, all patients received 1800 mg of FVP twice daily, and on the following day, 800 mg twice daily. FVP-induced liver damage was reported in three patients 13 (11–14) days after starting medication, which is statistically significant (*p* = 0.028). Patients who acquired FVP-induced liver impairment had higher serum concentrations than those who did not attend [32].

AST and ALT are two aminotransferases and are hepatocellular injury markers. AST is found in both cytosolic and mitochondrial isoenzymes, and it is in the liver and can also be elevated due to non-hepatic sources. ALT is a cytosolic enzyme present in the liver in high amounts. The release of these enzymes into the circulation is triggered by hepatocellular damage, not necessarily cell death [33].

A clinical study assessed Lopinavir/Ritonavir, Remdesivir, and FVP’s side-effects on the liver by monitoring aspartate aminotransferase, alanine aminotransferase, gamma glutamyl-transpeptidase, and alkaline phosphatase. A computerized tomography (CT) scan of the body area that contains the pancreas, stomach, intestines, liver, gallbladder, and other organs was conducted to rule out any cause of liver injury other than the antiviral drugs. The liver function parameter was evaluated before, during, and after admission, as well as before, during, and after discharge. In this trial, 36 patients were treated with Lopinavir/Ritonavir, 85 patients with Remdesivir, and 151 patients with FVP [34]. Patients with critical previous liver harm (persistent hepatitis with hepatocytes or severe cholestasis, liver cirrhosis, and liver metastases) were excluded from the study [34].

Antiviral treatment was proposed to an approximately equivalent number of females (139 patients) and males (133 patients), and the age of the patients enrolled in the research was between 26 and 88 years, with an average age of 60.18 years [33]. FVP treatment for COVID-19 patients revealed an increase in the level of hepato-renal markers [34]. The present study found that the most frequent chronic diseases in the patients in this study were DM (19.6%), HTN (13.0%), and both DM and HTN (13.5%). In alignment with the obtained results, a previous study noted that 32.9% of the patients had diabetes and 23% were hypertensive. They concluded that the FPV did not change the AST level dramatically, while it elevated GGT compared to other antivirals (*p* = 0.055). Regarding ALT, it was highly elevated during hospital admission (*p* = 0.025) and later after hospital clearance (*p* = 0.022) [34].

In another study [35], it was noted that FVP seriously elevates ALT levels and hydroxychloroquine by (13.9% and 2.6%, *p* < 0.0001, respectively). The AST level was also elevated (8.6% and 1.0%, *p* < 0.0001). These findings suggest that the FVP causes liver injury, a finding that agrees with the obtained results of the present study.

Our study showed a significant difference before and after FPV therapy according to AST, ALT enzyme, alkaline phosphatase, total bilirubin, and direct bilirubin in the treated group with FPV therapy. At the same time, all the patients in the untreated groups showed no significant difference for all variables. The increases expressed as a percentage of change (% change) in the levels of liver enzymes (AST and ALT), alkaline phosphatase, total bilirubin, and direct bilirubin in the treated groups were 137.18%, 72.17%, 34.73%, 94.59%, and 123.53%, respectively, and 4.71%, 8.05%, 6.80%, 78.67%, and 8.57% in the untreated group with FPV for all patients (*n* = 361). Patients with COVID-19 who were given remdesivir (RVP) and FVP in previous research showed an increase in hepato-renal markers. Both mean ± SD of ALT (70.65 ± 50.25) and AST (62.25 ± 25.46) were found to be higher in the RVP group compared to the FVP group. However, the FVP group had higher levels of both blood urea (56.67 ± 38.40) and serum creatinine (01.70 ± 02.41). Hematuria was absent in both groups and proteinuria was also unremarkable [36].

The liver damage caused by FVP may be the result of an individual’s unique response to the drug or its metabolites. We also suggested that the liver damage may result from a larger dose. Even with a high dose of FVP, there is a comfortable safety margin due to the large distance between the half-cytotoxic concentration (>400 M) and the half-maximal effective concentration (61.88 M) against SARS-CoV-2 [11]. Due to greater FVP plasma exposure in the Asian population, however, it is feasible for an elevated intracellular concentration to exceed the toxicity threshold [11,36]. This raises the possibility of regional or ethnic variations in its pharmacokinetics. Additionally, prolonged use inhibits its liver metabolism, which could increase the ratio of FVP to inactive metabolites. Furthermore, the previous report predicted a more than two-fold increase in FPV plasma concentrations over the half-maximal effective concentration [37].

A total of 194 adverse events were recorded by 93 patients, according to a previous study [12]. The most prevalent ADEs associated with FVP were elevated liver enzymes, upset stomach, palpitations, and diarrhea. Severe ADEs were more common in men and those over 64.

The present study found an increase in AST before and after FVP in patients 40–60 years of age and in patients over 60. For ALT, alkaline phosphatase, total bilirubin, and direct bilirubin, there were significant differences between the age groups of patients only after FPV treatment. Except for alkaline phosphatase in the under 40s and 40s and 60s, the results revealed significant differences between patients’ levels before and after FPV treatment.

The liver changes with age, which can affect clinical features and prognosis in people with liver disorders. As people get older, their liver volume and blood flow decline. In addition, drug metabolism may be harmed because of these alterations with decreased cytochrome P450 activity, raising the risk of drug-induced liver injury [38].

The processes of liver injury are intricate, and numerous pathways are involved in its regeneration processing [39]. It is possible that both cholestatic liver damage brought on by FVP and simultaneous liver damage brought on by other medications. In addition, severe COVID-19 cases have been linked to the risk of micro-thrombosis, which results in biliary obstruction [40]. Recently, it was shown that the angiotensin-converting enzyme 2 (ACE2) receptor is the entrance gateway for SARS-CoV-2 [9E11]. SARS-CoV-2 itself may have caused liver damage. In addition to the lung, the liver also expresses the ACE2 receptor, with the cholangiocytes expressing it more so than the hepatocytes [39,40]. To further explore the effects of FVP on liver function, more research with a larger sample size and the exclusion of patients with hepatic abnormalities before therapy are required.

In general, it was discovered that the enzymes increased significantly in patients after FPV treatment compared to patients before FPV for all age groups. The mean age is a confounding variable that increases all liver enzymes due to the decline in liver function with increasing age [41].

In our study, there were no statistically significant differences for all cases under investigation for all parameters (AST, ALT, alkaline phosphatase, total bilirubin, and direct bilirubin) in the patients who had chronic diseases and healthy cases. On the other side, no significant difference was found between before and after treatment for all parameters except for the patients’ AST. They do not have a chronic disease, and patients with chronic illness have elevated ALT and alkaline phosphatase.

This research has strong strengths. The study design is appropriate for the research question because the study population is large, and it is the first study in Saudi Arabia to assess liver injury caused by FVP. This study has some limitations, as a lack of references and including only the hospitalized patients in Jeddah city with different nationalities make it challenging to generalize the result to Saudis

## 5. Conclusions and Recommendations

The current study sought to investigate liver damage caused by FVP therapy. Results showed elevations in liver function enzymes after FPV use in COVID-19 patients. In general, liver enzymes increased significantly in patients of all ages after FPV treatment. According to all parameters, no statistically significant difference was found between healthy individuals and the chronic disease patients under study. A future randomized control trial is needed to prove or validate our results. To address the effects of Favipiravir on liver function further, another study with a larger sample size and the exclusion of individuals with hepatic abnormalities before medication is required.

## Figures and Tables

**Table 1:** Demographic data of the studied group.

	***n* (N = 362)**	**%**
	**Gender**	
Male	275	76.0%
Female	87	24.0%
	**Nationality**	
Saudi	98	27.1%
Non-Saudi	264	72.9%
	**Age**	
**ADULT** < 40	66	18.2%
**MIDDLE AGE** 40–60	204	56.4%
**ELDERLY** > 60	92	25.4%
**Mean (±SD)**	51.29 (±10.43)	
**Min–Max**	24–67	
	**Treated with FVP**	
Yes	181	50.0%
No	181	50.0%

**Table 2:** The effect of using another medication on liver enzymes.

	Medication	Mean ± SD	Mode	Median	t	sig
**AST. B**	Received drugs (*n* = 274)	46.99 ± 27.35	45	43	−1.086	0.278
	NA (*n* = 88)	50.69 ± 28.88	32	41		
**AST.A**	Received drugs	69.10 ± 93.51	50	50	−1.935	0.054
	NA	103.17 ± 240.16	29	44		
**ALT.B**	Received drugs	42.47 ± 25.83	20	36	−0.062	0.950
	NA	42.67 ± 26.28	29	36		
**ALT.A**	Received drugs	83.31 ± 163.69	35	54	−0.336	0.737
	NA	89.70 ± 121.23	28	51		
**Alkaline phosphatase** **. B**	Received drugs	27.59 ± 40.41	63	63	0.915	0.361
	NA	23.16 ± 35.76	58	58		
**Alkaline phosphatase** **. A**	Received drugs	34.19 ± 49.04	63	64	1.411	0.159
	NA	26.00 ± 40.49	8	61		
**Total bilirubin.** **B**	Received drugs	0.66 ± 6.15	0.31	0.50	0.609	0.543
	NA	0.25 ± 0.40	0.36	0.48		
**Total bilirubin** **. A**	Received drugs	0.44 ± 0.96	0.45	0.46	−0.128	0.898
	NA	0.45 ± 1.12	0.49	0.52		
**Direct** **bilirubin** **. B**	Received drugs	0.13 ± 0.19	0.20	0.26	1.513	0.131
	NA	0.09 ± 0.22	0.15	0.22		
**Direct** **bilirubin** **. A**	Received drugs	0.21 ± 0.54	0.21	0.26	−0.414	0.679
	NA	0.24 ± 0.74	0.13	0.25		

A: test used: unpaired *t*-test at *p* < 0.05. B: before treatment with medication for chronic disease; A: after treatment with medication for chronic disease; NA: no medications.

**Table 3:** Pearson correlation coefficient (r values, *p*-value) between the liver functions (before: above (normal); after: below (Bold)).

		AST	ALT	Alkaline Phosphates	Total Bilirubin	Direct Bilirubin
**AST**	r	1	0.440 **	0.106	0.075	0.240 **
	*p*-value		0.0001	0.155	0.320	0.001
**ALT**	r	**0.369** **	1	0.235 **	0.244 **	0.292 **
	*p* value	**0.000**		0.002	0.001	0.000
**Alkaline phosphates**	r	**0.899**	**0.286**	1	0.187 *	0.233 **
	*p* value	**0.009**	**0.080**		0.012	0.002
**Total bilirubin**	r	**0.259** **	**0.116**	**−0.023**	**1**	0.526 **
	*p* value	**0.000**	**0.120**	**0.754**		0.000
**Direct bilirubin**	r	**0.325** **	**0.129**	**−0.073**	**0.891** **	1
	*p* value	**0.000**	**0.087**	**0.334**	**0.000**	

Significant difference using *t*-test * at *p* < 0.05 and ** at *p* < 0.01.

**Table 4:** Comparison between before and after according to treatment with FVP.

Variables		Before	After	% Change	95% Confidence Interval		Paired. *t*-Test	*p* Values
					Lower	Upper		
**AST**	***Treated***	46.13 ± 4.49	109.41 ± 6.76	137.18%	−92.11	34.43	4.32	<0.001 **
	***Untreated***	47.38 ± 2.64	49.61 ± 3.58	4.71%	0.34	8.11	1.15	0.132 **
**ALT**	***Treated***	44.20 ± 5.21	123.63 ± 9.68	172%	−109.84	−49.02	5.15	<0.001 **
	***Untreated***	42.83 ± 6.53	46.28 ± 7.81	8.05%	−9.14	−1.75	1.61	0.061 *
**Alkaline phosphatase**	***Treated***	27.90 ± 4.29	37.59 ± 5.29	34.73%	−13.86	−5.52	4.58	<0.001 **
	***Untreated***	25.15 ± 6.42	26.86 ± 8.29	6.80%	−2.81	−0.59	1.24	0.360
**Total bilirubin**	***Treated***	0.37 ± 0.33	0.72 ± 0.37	94.59%	−0.54	−0.17	3.83	<0.001 **
	***Untreated***	0.75 ± 0.51	0.16 ± 0.15	78.67%	−0.52	1.69	1.05	0.295
**Direct bilirubin**	***Treated***	0.17 ± 0.21	0.38 ± 0.24	123.53%	−0.32	−0.11	4.03	<0.001 **
	***Untreated***	0.07 ± 0.01	0.064 ± 0.11	8.57%	−0.001	0.02	1.73	0.085

* Significant difference using paired *t*-test at *p* < 0.05 and ** at *p* < 0.01; Treated: treated with FVP; Untreated: untreated with FVP; Before: baseline of liver enzyme level; After: liver enzyme after treatment with FVP in case-treated or Saudi MOH protocol for COVID-19 management in case-untreated.

**Table 5:** Comparison between treated and untreated after FPV Therapy.

Variables	Untreated	Treated	% Change	95% Confidence Interval		Unpaired *t*-Test	*p* Values
				Lower	Upper		
**AST** **(5–34 U/L)**	45.38 ± 7.24	109.41 ± 6.76	141.1%	35.02	93.01	4.34	<0.001 **
**ALT** **(5–55 U/L)**	46.28 ± 7.81	123.63 ± 9.68	167.13%	46.36	108.32	4.91	<0.001 **
**Alkaline phosphatase** **(40–150 U/L)**	26.86 ± 8.29	37.59 ± 5.29	39.95%	1.01	20.45	2.17	0.031 **
**Total bilirubin** **(0.2–1.2 mg/dL)**	0.16 ± 0.15	0.72 ± 0.37	350.0%	0.36	0.76	5.57	<0.001 **
**Direct bilirubin** **(0.1 to 1.2 mg/dL)**	0.24 ± 0.11	0.38 ± 0.24	58.33%	0.19	0.43	5.22	<0.001 **

** means significant difference using paired *t*-test at *p* < 0.05.

**Table 6:** A chronic disease with inpatients treated and non-treated with FVP.

Parameters	Chronic Disease	Before (*n* = 181)	After (*n* = 181)	*t*-Test	Sig
**AST** **(5–34 U/L)**	Yes (*n* = 222)	45.11	76.11	**2.87**	**0.004** **
	No (*n* = 140)	50.34	71.25	**2.901**	**0.004** **
	***t*-test**	**2.44**	**0.65**		
	**Sig**	**0.015** **	**0.948**		
**ALT** **(5–55 U/L)**	Yes	42.45	85.52	**3.53**	**<0.001** **
	No	42.18	86.63	**5.975**	**<0.001** **
	***t*-test**	**0.05**	**0.260**		
	**Sig**	**0.956**	**0.795**		
**Alkaline phosphatase** **(40–150 U/L)**	Yes	27.54	34.24	**4.18**	**<0.001** **
	No	22.99	27.13	**3.075**	**0.003** **
	***t*-test**	**1.167**	**1.45**		
	**Sig**	**0.550**	**0.421**		
**Total bilirubin** **(0.2–1.2 mg/dL)**	Yes	0.76	0.39	**0.763**	**0.04** **
	No	0.26	0.46	**2.31**	**0.023** **
	***t*-test**	**0.842**	**1.141**		
	**Sig**	**0.400**	**0.842**		
**Direct bilirubin** **(0.1 to 1.2)**	Yes	0.13	0.19	**2.454**	**0.015** **
	No	0.11	0.24	**2.861**	**<0.001** **
	***t*-test**	**0.843**	**1.16**		
	**Sig**	**0.400**	**0.247**		

** means significant difference using paired *t*-test at *p* < 0.05.

## Data Availability

The collected data from the hospital information system (careware) were added to an Excel datasheet. Patients with COVID-19 were studied at Jeddah’s East Jeddah hospital from May to August 2020.

## Abbreviations

AST	aspartate transaminase
ADEs	adverse events
ALT	alanine transaminase
FVP	favipiravir
GGT	gamma-glutamyl transferase
ICU	intensive care unit
